# Physiologically Based Pharmacokinetic Model Development and Verification for Bioequivalence Testing of Bempedoic Acid Oral Suspension and Reference Tablet Formulation

**DOI:** 10.3390/pharmaceutics15051476

**Published:** 2023-05-12

**Authors:** Benny M. Amore, Nikunjkumar Patel, Priya Batheja, Ian E. Templeton, Hannah M. Jones, Michael J. Louie, Maurice G. Emery

**Affiliations:** 1 Esperion Therapeutics, Inc., Ann Arbor, MI 48108, USA; pbatheja@esperion.com (P.B.); mlouie@esperion.com (M.J.L.); memery@esperion.com (M.G.E.); 2 Certara, Inc., Princeton, NJ 08540, USA; nikunjkumar.patel@certara.com (N.P.); ian.templeton@certara.com (I.E.T.); hannah.jones@certara.com (H.M.J.)

**Keywords:** bempedoic acid, bioequivalence, pharmacokinetics, physiologically based pharmacokinetic model

## Abstract

The bioequivalence of bempedoic acid oral suspension and commercial immediate release (IR) tablet formulations were assessed using a physiologically based pharmacokinetic (PBPK) model. The mechanistic model, developed from clinical mass balance results and in vitro intrinsic solubility, permeability, and dissolution data, was verified against observed clinical pharmacokinetics (PK) results. Model inputs included a fraction of a dose in solution (0.01%), viscosity (118.8 cps), and median particle diameter (50 µm) for the suspension and particle diameter (36.4 µm) for IR tablets. Dissolution was determined in the relevant media (pH 1.2–6.8) in vitro. Model simulations of bioequivalence predicted oral suspension (test) to IR tablet (reference) geometric mean ratio estimates of 96.9% (90% confidence interval [CI]: 92.6–101) for maximum concentration and 98.2% (90% CI: 87.3–111) for the area under the concentration–time curve. Sensitivity analyses showed gastric transit time had a minor impact on model predictions. Oral suspension biopharmaceutical safe space was defined by extremes of particle size and the percent of bempedoic acid in solution. PBPK model simulations predicted that the rate and extent of bempedoic acid absorption are unlikely to exhibit clinically meaningful differences when dosed as an oral suspension compared with an IR tablet without requiring a clinical bioequivalence study in adults.

## 1. Introduction

Bempedoic acid significantly reduces low-density lipoprotein cholesterol (LDL-C) in adults with hypercholesterolemia [1]. The mechanism by which LDL-C levels are reduced involves the activation of bempedoic acid to a coenzyme A thioester (ETC-1002-CoA) by a very long-chain acyl-CoA synthetase 1 (ACSVL1), an enzyme primarily expressed in the human liver and kidney but not in skeletal muscle or most other peripheral tissues. The pharmacologically active ETC-1002-CoA inhibits ATP citrate lyase, a precursor to 3-hydroxy-3-methylglutaryl coenzyme A (HMG-CoA) reductase in the pathway of cholesterol biosynthesis, and subsequently leads to LDL receptor upregulation and decreases in LDL-C levels [2].

As a weak acid, bempedoic acid is characterized by poor aqueous solubility at low pH and high passive permeability. Although these physicochemical properties align with Biopharmaceutical Classification System (BCS) class two (low solubility and high permeability) drugs [3], bempedoic acid is soluble at the pH conditions of the small intestine and is readily absorbed, as evidenced by peak plasma concentrations (C_max_) observed approximately 3.5 h after oral tablet administration. The clinical pharmacokinetics (PK) of bempedoic acid are linear, with dose-proportional increases in systemic exposure observed over a range of 120 to 220 mg/day. Bempedoic acid PK is characterized by an elimination half-life of approximately 21 h, a moderate volume of distribution that is less than total body water, high binding to plasma proteins, and an accumulation ratio of approximately 2.3-fold when dosed daily [4]. Hepatic metabolism represents the main pathway of elimination, where bempedoic acid is primarily metabolized by uridine 5′ diphospho-glucuronosyltransferase (UGT) 2B7 to form an inactive acyl glucuronide metabolite. Bempedoic acid also undergoes reversible metabolism to form a keto metabolite, ESP15228, that becomes activated to a coenzyme A thioester by ACSVL1 and inhibits ATP citrate lyase with similar potency to ETC-1002-CoA [5].

Bempedoic acid is being evaluated as a potential treatment for hyperlipidemia in pediatric populations. An initial phase 2 study in children aged 6–17 years with heterozygous familial hypercholesterolemia (HeFH) was designed to evaluate the PK, pharmacodynamics, and safety of bempedoic acid (NCT05694260). For children who are unable to ingest a solid tablet dosage form, an oral suspension dosing formulation of bempedoic acid was developed and a first evaluation in pediatric subjects was planned for the phase 2 trial. Determination of the relative bioavailability of a new formulation is generally required prior to first use in pediatric subjects and is frequently accomplished by conducting a human bioequivalence trial in adults. However, the US Food and Drug Administration (FDA) may grant a biowaiver request supported by physiologically based pharmacokinetic (PBPK) modeling when accompanied by suitable in vitro evaluations that provide model input to predict drug absorption [6].

Objectives of this study included the development of a PBPK model to simulate bempedoic acid plasma concentrations and predict oral absorption based on suspension and tablet dissolution determined under comparable in vitro conditions. A final model was applied to evaluate the bioequivalence of oral suspension and immediate release (IR) tablet formulations of bempedoic acid 180 mg in a virtual population of healthy adults. The PBPK model was verified against observed bempedoic acid PK data from completed clinical studies. Model sensitivity to the potential impact of inter-individual variability was explored with respect to gastrointestinal physiology and differences between oral suspension and tablet formulations. Based on PBPK model simulations, a biowaiver was granted by the FDA for the bempedoic acid suspension, negating the need for a clinical bioequivalence trial in adult subjects. To the best of our knowledge, this is the first use of PBPK modeling in the development and regulatory assessment of a pediatric formulation.

## 2. Materials and Methods

### 2.1. Materials

Bempedoic acid clinical data was derived from previous studies conducted with [^14^C] bempedoic acid 240 mg formulated as an oral solution (Lot 60309-13-001), a 180 mg tablet used during clinical development (multiple lots), or the 180 mg commercial tablet (multiple lots). Film-coated IR tablets were manufactured by wet granulation followed by drying, lubrication, and compression into tablets.

Bempedoic acid oral suspension (20 mg/mL; Lot 0000091928) used in the present study was prepared at a pilot scale (100 L) in support of development of a clinical formulation to be tested in pediatric subjects.

### 2.2. Clinical Studies

PBPK model development and verification were based on bempedoic acid PK results from four phase 1 clinical trials in adult subjects (Table 1). A PBPK base model was initially developed from the results of a human absorption, distribution, metabolism, and excretion study in healthy subjects who received radiolabeled bempedoic acid (Study 001). Bempedoic acid PK were determined in 6 adult male subjects aged 33 to 59 years with blood collections up to 168 h after receiving a single oral solution dose of [^14^C] bempedoic acid 240 mg.

Verification of the PBPK base model was achieved by comparing model predictions with observed bempedoic acid PK following single-dose (Study 002) and multiple-dose (Study 003) administrations of bempedoic acid 180 mg. Study 002 assessed the bioequivalence of two clinical development IR tablet formulations of bempedoic acid in an open-label, 2-sequence crossover study in 58 healthy subjects (aged 19–56 years, 34% female). Single-dose PK verification of the PBPK base model was determined by comparing model predictions to observed PK of the reference tablet (Formulation 1) in Study 002. Study 003 was an open-label, single-sequence study in 48 healthy subjects to evaluate the potential for PK interactions between concomitant dosing of four statins (atorvastatin, simvastatin, pravastatin, and rosuvastatin) when taken alone (reference treatments) and in combination with steady-state bempedoic acid (test treatments). Each cohort included 12 subjects who received combined treatments of bempedoic acid and atorvastatin (aged 20–58 years, 33% female), simvastatin (aged 18–57 years, 8% female), pravastatin (aged 19–55 years, 50% female), or rosuvastatin (aged 19–56 years, 23% female). Verification of the PBPK base model predictions of multiple-dose PK was assessed against observed PK of bempedoic acid from each of the 4 drug–drug interaction (DDI) cohorts following 12 days of 180 mg/day dose administration.

Study 004 was conducted in 59 healthy subjects (aged 20–60 years, 57% female) to assess the bioequivalence of two IR tablet formulations of bempedoic acid in an open-label, 2-sequence crossover of Formulation 2 (test) and Formulation 1 (reference) bempedoic acid 180 mg tablets where Formulation 1 is a tablet used during clinical development and Formulation 2 is the commercial tablet formulation of bempedoic acid. On Day 1, after an overnight fast, subjects were randomized to receive a single oral dose of bempedoic acid 180 mg as Formulation 2 or Formulation 1. On Day 15, subjects were crossed over to receive an oral dose of the second tablet formulation following an overnight fast. Serial PK blood samples were collected over a period of 120 h following dose administration with a washout period of 14 days between treatments. Comparisons of geometric least square mean parameter ratios of Study 004 concluded no significant differences in the rate and extent of absorption between the commercial tablet (Formulation 2) and the reference tablet (Formulation 1) shown in Table S1. Elements of the study design and demographic profiles of individual subjects from Study 004 were used to select the size of a virtual subject population required to adequately assess bioequivalence and to develop simulation conditions for the virtual trial evaluating the commercial IR tablet and oral suspension formulations.

In each of the 4 clinical studies, bempedoic acid plasma concentrations were determined by liquid chromatography-tandem mass spectrometry (LC-MS/MS) as described previously [7]. All trials were conducted in compliance with the ethical principles of the Declaration of Helsinki and with the International Conference on Harmonization Good Clinical Practice guidelines, and all subjects provided written informed consent prior to study participation. Study protocols and informed consent documents of each study were reviewed and approved by the appropriate institutional review board or ethics committee.

### 2.3. In Vitro Solubility and Dissolution

Equilibrium solubility of a saturated solution of bempedoic acid drug substance was determined in common dissolution buffers across a pH 1.0 to pH 6.8 range. Saturated buffer solutions were prepared with excess bempedoic acid followed by incubation at 37 °C.

Comparative dissolution of the IR tablet (180 mg; Lot 99743-07D) and oral suspension (180 mg) formulations were performed under identical experimental conditions. Dissolution tests were performed using a calibrated USP II (paddle) apparatus maintained at 37 °C. Each vessel contained 900 mL of biorelevant dissolution media, and the rotational speed was set at 50 rpm. Dissolution of the IR tablet and oral suspension was determined at pre-specified timepoints at 5, 10, 15, 20, 30, 45, 60, and 75 min in relevant media that represented pH conditions of the gastrointestinal tract. Dissolution media included 0.1N hydrochloric acid (HCl; pH 1.2), 50 mM sodium acetate buffer (pH 4.5), and 50 mM potassium phosphate buffer (pH 6.6 and pH 6.8).

Bempedoic acid concentrations were determined in samples collected from equilibrium solubility and dissolution experiments by liquid chromatography (LC). Solubility sample quantitation was achieved by high-performance LC (HPLC) on an Agilent Zorbax C18 column (50 × 3 mm, 1.8 µm) with UV detection at 215 nm. Analyte separation was achieved by gradient elution with mobile phase (0.05% trifluoracetic acid [TFA] in water, *v*/*v* and 0.05% TFA in acetonitrile, *v*/*v*) at a constant flow rate of 1.5 mL/min. Bempedoic acid quantitation in tablet dissolution samples was determined by HPLC using a Luna phenyl hexyl 100 A LC column (150 × 4.6 mm, 5 µm; Phenomenex) with UV detection at 210 nm. Analyte separation was achieved by isocratic elution with mobile phase (acetonitrile:phosphate buffer [pH 3]; 55:45) at a constant flow rate of 1.0 mL/min. Suspension dissolution samples were analyzed by ultra-performance LC using a Waters XBridge C18 column (150 × 4.6 mm, 3.5 µm) with UV detection at 215 nm. Analyte separation was achieved by isocratic elution with mobile phase (0.05% formic acid in acetonitrile:water 60:40 *v*/*v*) at a constant flow rate of 1.0 mL/min. 

### 2.4. Modeling Strategy

A mechanistic PBPK model was used to conduct virtual trials for the comparison of PK predictions when bempedoic acid is dosed as either an oral suspension or an IR tablet. The distribution and elimination of bempedoic acid were represented by a minimal PBPK model structure that included the gut, liver, and portal vein with combined distribution to all other tissues represented as a single adjusting compartment (Figure S1). Predictions of bempedoic acid absorption kinetics employed the advanced dissolution absorption and metabolism (ADAM) model within the Simcyp PBPK simulator, where a catenary series of compartments representing sub-regions of the gastrointestinal tract provides estimates of the rate and extent of bempedoic acid absorption. Drug absorption from each segment was described as a function of dissolution, precipitation, luminal degradation, permeability, and transit between segments. A diffusion layer model (DLM) within the PBPK simulator predicts particle dissolution and was used to differentiate between liquid suspension and IR tablet dosage forms. The model assumes a spherical particle surface and non-linear diffusion of dissolved drugs [8]. The DLM input parameters for the suspension included bempedoic acid concentration, measured viscosity of the suspension, measured particle radius, and estimated fraction of dissolved drug based on bempedoic acid solubility at pH of suspension. For the IR tablet, measured particle radius was entered directly. DLM predictions of particle dissolution were assessed by comparing against experimental in vitro dissolution data for the IR tablet and suspension. All simulations were conducted using Simcyp PBPK Simulator V18 (Certara UK Limited, Sheffield, UK; release 2; 18.1.122.0). Simulated PK data from the Simcyp V18 virtual bioequivalence module were transferred to the Phoenix platform where bioequivalence assessments were performed using Phoenix 64 (Certara, Inc., Princeton, NJ, USA; v8.1.0.3530).

### 2.5. Model Development and Verification

The base PBPK model was developed by verifying PK parameters (absorption and clearance) from a bempedoic acid mass balance study (Study 001), in vitro characterizations of intrinsic solubility, plasma protein binding, blood–plasma partitioning, and model-defined parameters related to permeability, intercompartmental clearance, and volume of distribution.

Oral clearance (CL_oral_) and renal clearance (CL_renal_) were determined in healthy adult subjects after [^14^C] bempedoic acid 240 mg dosing were 0.81 L/h and 0.03 L/h, respectively. Bempedoic acid was well absorbed with predicted 0.97 fraction of dose absorbed (f_a_) based on passive permeability in Caco-2 cells. The ratio of blood to plasma concentration (B:P) of radioactivity was determined in clinical samples after [^14^C] bempedoic acid 240 mg dosing (Study 001). B:P ratio was set to 0.55 in the model, the lowest value permitted in the simulator, consistent with experimentally derived B:P for area under the concentration–time curve (AUC; 0.498) and maximum concentration (C_max_; 0.489) from Study 001 samples. Bempedoic acid protein binding in human plasma was determined by ultrafiltration with radiochemical detection at PharmOptima LLC (Portage, MI, USA). Protein binding (2.6% unbound) was estimated from bempedoic acid at 3 and 10 µg/mL incubations to approximate protein binding within the concentration range observed clinically. Intrinsic solubility was estimated at 0.0051 mg/mL.

Bempedoic acid steady-state volume of distribution (V_ss_) was predicted according to Equation (1) [9]:

(1) $$V_{ss}=\left(\sum V_{t}\times P_{t:p}\right)+\left(V_{e}\times E:P\right)+V_{p}.$$

In the above equation, V is the fractional body volume (L/kg) of tissue (t), erythrocyte (e), and plasma (p), and E:P is the erythrocyte:plasma ratio. A tissue-to-plasma partition coefficient (P_t:p_) was predicted using mechanistic equations that account for drug ionization [10,11].

Bempedoic acid unbound hepatic intrinsic clearance (CL_u,int,H_) was predicted according to Equation (2):

(2) $${CL}_{u,int,H}=\frac{\frac{{CL}_{oral}}{B:P}\times f_{a}\times F_{G}-\frac{{CL}_{renal}}{B:P}}{Uptake\times {fu}_{b}\times \left(1+\frac{\frac{{CL}_{renal}}{B:P}}{Q_{H}}\right)}.$$

In the above equation, CL_oral_ is the observed oral clearance, B:P is the concentration ratio of drug in blood to plasma, fu_b_ is fraction of unbound drug in blood (calculated from f_up_/B:P), Q_H_ is the blood flow in the hepatic vein (90 L/h), F_G_ is fraction escaping first-pass metabolism in the gut (assumed to be 1), CL_renal_ is the renal clearance, Uptake accounts for hepatic active uptake (set to value of 1), and f_a_ is fraction of dose absorbed.

Verification of the base model was achieved by comparing bempedoic acid PK predictions against observed results following bempedoic acid administration as an IR tablet. The predictive performance of the PBPK base model was assessed by comparing model-predicted bempedoic acid PK parameters, derived from 10 virtual trials per simulation, against observed clinical PK parameters following a single dose of bempedoic acid (Study 002) and repeated daily dose (Study 003) administration of a 180 mg IR tablet. Additionally, simulated data were visually inspected against observed data, where observed concentration–time data were confirmed to fall within the 5th and 95th percentile prediction intervals of simulated data. Sample size, age range, and proportions of male and female subjects used in the simulations were matched to the subject demographics of each clinical study. Sensitivity analyses were conducted to assess the potential impact of gastrointestinal physiological parameters on model predictions of bempedoic acid absorption, including stomach pH and gastric, small intestine, and colonic transit times.

Predictions of bempedoic acid PK following administration as an oral suspension were simulated using a verified IR tablet PBPK model that included parameters for the oral suspension. A diffusion model within the PBPK simulator was used to predict particle dissolution for each formulation. Unique DLM scalars derived from estimates of bempedoic acid concentration, particle radius, viscosity, and fraction dissolved in solution for the suspension and particle radius for the IR tablet were incorporated into the PBPK model. Verification of the final model included comparisons of model predictions of dissolution versus in vitro experimental dissolution data for the IR tablet and suspension.

### 2.6. Model Application

The potential for population size to impact the assessment of oral suspension and IR tablet bioequivalence using the PBPK final model was evaluated by comparing model predictions to observed bempedoic acid PK from the bioequivalence trial. In Study 004, the sample size was calculated using an overall power of 80% or greater with an alpha error of 5%, where power was defined as the probability of the 90% confidence interval (CI) of the test/reference ratio being within the acceptance criteria of 80% to 125%. The PBPK final model was evaluated by matching the sample size and demographic profile of study subjects from the bioequivalence trial to run initial model simulations where a total of 10 trials were conducted using a population of 59 virtual subjects per trial with age, sex, height, and body weight matched to the parameters of individual subjects who participated in Study 004. Physiological parameters related to absorption were generated randomly from population distributions defined within the PBPK simulator. Virtual population size for bioequivalence assessment was confirmed by comparing observed PK exposures from the reference population of Study 004 with predicted bempedoic acid PK from each of 10 trial simulations. Sensitivity analyses were conducted to assess the impact of physiological parameter estimates on model predictions of bempedoic acid PK. Subsequent assessments of commercial IR tablet (reference) and oral suspension (test) bioequivalence in virtual subjects after bempedoic acid 180 mg administration were conducted in crossover and parallel group studies. Sensitivity analyses of the virtual trials were conducted by assuming a boundary condition (worst-case) of complete bempedoic acid dissolution in the oral suspension preparation and assessing the impact on bioequivalence determinations.

## 3. Results

### 3.1. Aqueous Solubility and Dissolution

Bempedoic acid is a dicarboxylic acid with dissociation constants of pKa_1_ at 4.88 and pKa_2_ at 5.60. Aqueous solubility studies in buffered media revealed that bempedoic acid exhibited pH-dependent solubility, with low solubility below pH 6 and greatly increased solubility above pH 6. In vitro dissolution was determined for the IR tablet (180 mg) and oral suspension (180 mg) formulations in biorelevant media conditions of 0.1N HCl, pH 1.2; 50 mM acetate buffer, pH 4.5; 50 mM phosphate buffer, pH 6.6, and pH 6.8. Consistent with observations of pH-dependent solubility, the extent of dissolution of both the IR tablet (Table S2) and oral suspension (Table S3) was limited to low pH conditions in HCl and acetate media. In contrast to lower-pH media conditions (pH ≤ 4.5), the extent of dissolution was near complete for each solid formulation in the pH range of the human intestine (pH > 6).

### 3.2. PBPK Base Model

Bempedoic acid distribution and elimination were represented by a minimal PBPK model structure that included gut, liver, and portal vein with combined distribution to the remainder of tissues represented as a single-adjusting compartment (Figure S1). Model parameters were further refined by fitting the base model to observed clinical PK data from healthy subjects in Study 001. Observed bempedoic acid plasma concentration–time data were within the 90% CI of predicted PK from 10 virtual trial simulations comprised six fasted virtual healthy male subjects each. Ratios of predicted to observed geometric mean estimates for C_max_ (0.86), AUC from zero to infinity (AUC_inf_, 1.06), elimination half-life (1.07), and median time to maximum concentration (t_max_, 1.46) confirmed the initial suitability of the base model.

**Figure 1:**
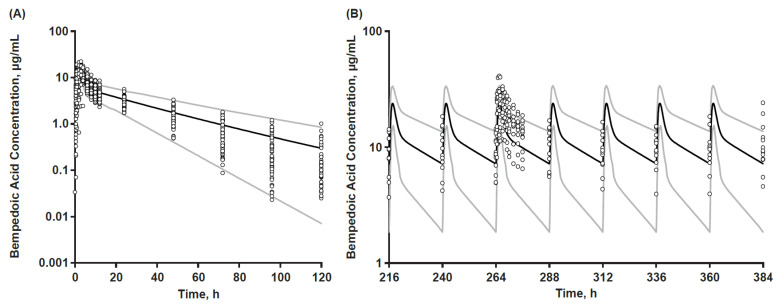
Base model verification. Bempedoic acid population mean simulated (black line) with 5th and 95th percentiles (grey lines) and individual observed plasma concentration–time data (open symbols) after (**A**) Single and (**B**) Multiple bempedoic acid 180 mg dosing of studies 002 and 003 (bempedoic acid-atorvastatin cohort, 12 subjects), respectively.

Verification of the base model was subsequently achieved by comparing model predictions against clinical data sets that were distinct from Study 001 used in model development. The predictive performance of the PBPK-base model was assessed against observed clinical PK following bempedoic acid single-dose (Study 002) and multiple-dose (Study 003) administrations as a 180 mg IR tablet (Figure 1). 

Steady-state concentration–time data from each of the four DDI cohorts in Study 003 were treated as separate data sets when compared with model predictions of bempedoic acid PK exposure. Simulations were conducted in virtual populations with numbers of subjects, ages, and female-to-male ratios matched to each clinical study population. Bempedoic acid-base model predictions were within 0.52- to 1.33-fold of observed data after single-dose administration in Study 002 and within 0.77- to 1.11-fold of observed data after 12 daily oral doses of 180 mg IR tablet across the four cohorts of Study 003 (Table 2).

### 3.3. Final Model

Predictions of bempedoic acid PK following administration of an oral suspension were derived from a PBPK model of bempedoic acid in which the base model was modified with input parameters specific to both the suspension and IR tablet. The modified PBPK model (final model) incorporated estimates of bempedoic acid concentration, particle radius, viscosity, and fraction dissolved in solution corresponding to the oral suspension and particle radius for the IR tablet (Table 3). The final model was established by using the DLM within the PBPK simulator to predict particle dissolution in order to differentiate the two oral dosage forms of bempedoic acid based on the rate and extent of dissolution. DLM input parameters for the oral suspension prepared at pH 4.53 included a bempedoic acid concentration of 20 mg/mL and a median API particle diameter of 50 µm. The viscosity of the suspension was determined to be 118.8 centipoise (cps). The estimated viscosity was included in the analysis of in vitro dissolution using the modeling platform, SIVA, and for in vivo simulations of the stomach segment using the Simcyp simulator. Hence, the viscosity of the dissolution medium in vitro as well as stomach fluid in vivo were adjusted to 118.8 cps, and the diffusivity of molecules around the dissolving particle surface was calculated using this viscosity estimate. The fraction of bempedoic acid in solution was assumed to be negligible (0.01%) in the suspension formulation, based on low solubility at pH 4.53 and >99.9% undissolved drug estimated for a dose of 180 mg. The suspension did not contain excipients to increase solubility or prevent precipitation. Surfactant was added at low concentrations as a wetting agent but is not expected to have impacted the fraction of bempedoic acid in the solution. An estimated median API particle diameter of 36.4 µm for the IR tablet was used in the final model.

**Figure 2:**
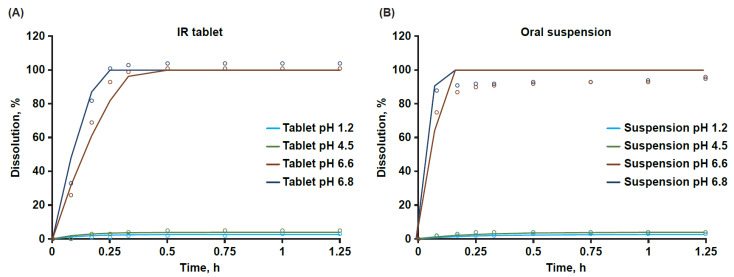
Observed and predicted dissolution of bempedoic acid IR tablet (**A**) and oral suspension (**B**). In vitro dissolution determined in USP II apparatus (paddle apparatus) with 900 mL of relevant dissolution media (0.1 N hydrochloric acid, pH 1.2; 50 mM acetate buffer, pH 4.5; 50 mM phosphate buffer, pH 6.6, and pH 6.8) stirred at 50 rpm at 37 °C. Symbols represent observed dissolution data, and solid lines represent model predictions. Lines and symbols are shown for pH 1.2 (blue), pH 4.5 (green), pH 6.6 (brown), and pH 6.8 (aqua) test conditions. IR, immediate release.

In vitro dissolution of the IR tablet (180 mg) and oral suspension (180 mg) were determined under identical conditions in biorelevant media. Verification of the model included comparisons of DLM predictions of dissolution versus in vitro experimental dissolution data for the IR tablet and oral suspension (Figure 2). At early timepoints, the predicted dissolution profiles indicated a slightly slower dissolution rate for tablet than for suspension, likely due to disintegration processes that occurred for the tablet but not for the suspension. At later timepoints, model predictions of IR tablet dissolution in phosphate buffer approximated complete dissolution observed for the tablet. However, predictions of suspension dissolution in phosphate buffer at the same experimental pH conditions were slightly greater than the observed data, where approximately 93% to 96% dissolution was measured in vitro at late timepoints. The overprediction of suspension dissolution is possibly due to the use of a method developed for the pilot batch suspension that was not optimized. Tablet dissolution results were generated with an optimized commercial dissolution method, and 100% bempedoic acid release would be anticipated with a fully optimized, phase-appropriate method for a final suspension product. Based on the resulting simulations in the various relevant media conditions, the PBPK model was considered fit-for-purpose to differentiate IR tablet and oral suspension performance.

### 3.4. Determination of Virtual Population Sample Size

**Figure 3:**
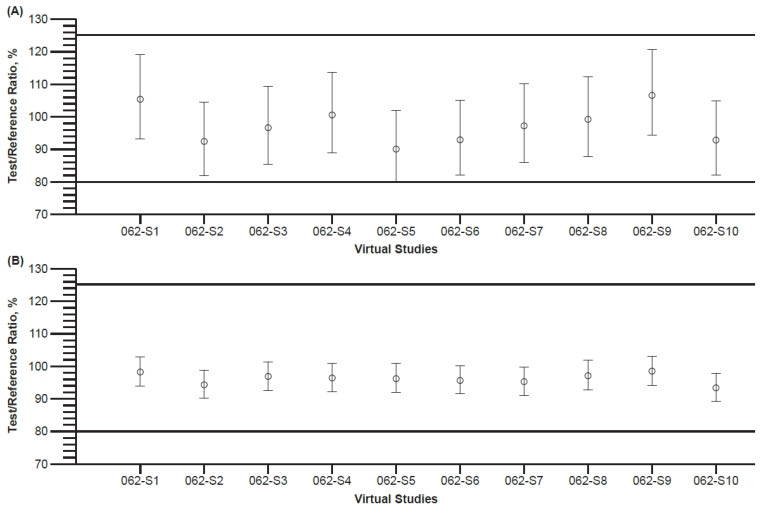
Geometric least square mean ratios (90% CI) of bempedoic acid (**A**) AUC_inf_ and (**B**) C_max_ parameter predictions (Test) from 10 virtual trials (Virtual Studies 062-S1 to 062-S10) and observed PK estimates from Study 1002-004 (Reference) for 180 mg doses of bempedoic acid formulated as an IR tablet. Range of 80% to 125% for bioequivalence (horizontal lines) shown. AUC_inf_, area under the concentration–time curve from time zero to infinity; C_max_, maximum concentration; CI, confidence interval; IR, immediate release; PK, pharmacokinetics.

PBPK final model simulations were conducted to investigate the impact that sample size might have on bioequivalence assessments of the IR tablet and oral suspension. The sample size was evaluated in initial model simulations of bempedoic acid dosed as an IR tablet. Simulations were conducted in 10 virtual trials and each trial comprised a population of 59 healthy subjects matched to the age, sex, body weight, and height of individual subjects in the bioequivalence trial (Study 004). Predictions of bempedoic acid PK from each of the 10 virtual trials were compared with observed PK results from subjects who received commercial IR tablet dosing in Study 004 to assess the adequacy of sample size. Observed clinical PK were well described by the PBPK final model and confirmed that predictions of C_max_ and AUC_inf_ exposures were representative of clinical bempedoic acid PK following administration of the commercial IR tablet. Ratios of simulated PK exposure parameters and observed C_max_ and AUC_inf_ estimates were within the 0.8 to 1.25 boundaries of bioequivalence in the ten virtual trials except for one trial where the lower edge of the 90% CI for AUC_inf_ fell below 0.8 (Figure 3). These findings supported the selection of a sample size of 59 healthy subjects to conduct virtual BE trials comparing the IR tablet and oral suspension formulations.

### 3.5. Final PBPK Model Sensitivity Analyses 

Although demographic characteristics of the virtual population were matched to subjects in the clinical trial, physiological parameters related to drug absorption were generated randomly from population distributions defined within the PBPK simulator. Sensitivity analyses were conducted to assess the potential for uncertainties in model parameters of gastrointestinal physiology to impact C_max_, t_max_, and AUC_inf_ predictions. Model sensitivity was determined by the correlation of physiological parameter estimates (stomach pH under fasted conditions and gastric transit times in the stomach, small intestine, and colon) and predictions of C_max_, t_max_, and AUC from simulations of 10 virtual trials with 59 subjects per trial. These sensitivity analyses demonstrated that gastric transit time had a minor impact on model predictions of the rate of absorption but not the extent of absorption when bempedoic acid is dosed as either a suspension or IR tablet. Longer residence time in the stomach was associated with lower C_max_ (r = −0.32) and a later time of peak concentration (t_max_ r = 0.40) predictions (Table 4). By contrast, bempedoic acid PK predictions of C_max_, t_max_, and AUC_inf_ were minimally impacted by variations in gastric pH and intestinal and colonic transit times.

### 3.6. Final Model Application in Virtual Bioequivalence Assessment

A final PBPK model was used to simulate bempedoic acid PK and assess the bioequivalence of single doses of bempedoic acid 180 mg administered as an oral suspension and commercial IR tablet. Virtual crossover and parallel trials were conducted in a population of 59 virtual subjects aged 20 to 60 years with equal proportions of male and female subjects.

A virtual crossover bioequivalence trial between suspension and tablet predicted geometric mean ratio (90% CI) estimates of 99.7% (96.1–103%) and 100% (90.6–110%) corresponding to C_max_ and AUC to time of last quantified concentration (AUC_last_) exposures, respectively. Model simulations from a virtual parallel bioequivalence trial predicted geometric mean ratio (90% CI) estimates of 96.9% (92.6–101%) and 98.2% (87.3–111%) corresponding to C_max_ and AUC_last_ exposures, respectively (Table 5). Differences in bempedoic acid C_max_ and AUC exposures between the pilot scale suspension and commercial IR tablet dosing are not expected to be clinically meaningful as geometric mean ratios and associated CIs were within the 80% to 125% range of bioequivalence in the crossover and parallel virtual trial designs.

### 3.7. Sensitivity Analysis (Worst Case) of Virtual Bioequivalence Predictions

Sensitivity analyses of the final model were conducted to determine the safe space for bioequivalence between formulations. Key parameters of the suspension formulation considered for evaluation included the fraction of dose in solution as well as particle size and viscosity, both of which were measured experimentally. Thus, analyses were conducted to determine how the fraction of bempedoic acid dissolved in the oral suspension might impact the assessment of suspension and tablet bioequivalence, where the fraction of dissolved drug in the oral suspension formulation prepared at pH 4.53 was estimated to be 0.01% in the final model (Table 3). To test model sensitivity, simulations of tablet bioequivalence were conducted with an oral suspension at a boundary parameter value of greater than 99% dissolved bempedoic acid as this represented the maximal possible difference from the reference tablet formulation where 100% of the dose is a solid (i.e., 0% dissolved). Model simulations of a suspension with a large fraction of bempedoic acid in solution did not reveal differences in a virtual crossover or parallel trial outcomes, with geometric mean ratios of PK parameter estimates bounded by the 0.8 to 1.25 limits of bioequivalence for the suspension and commercial tablet formulations. At the lower pH of the stomach, an increased fraction of the dose in solution for the suspension might impact initial rates of dissolution in the stomach, causing a potential shift in t_max_ relative to the tablet. However, when 99% of the dose in solution was assumed for the suspension, there was negligible impact on t_max_. Given the population variability in gastric emptying rates, the overall effect of the fraction of dissolved dose on bempedoic acid bioavailability was negligible.

## 4. Discussion

To support the planned investigations of bempedoic acid in pediatric patients with HeFH, an oral suspension formulation is being developed for children younger than eight years old and for those who have difficulty swallowing an adult tablet. Before evaluating the oral suspension in a pediatric Phase 2 clinical trial, the modeling and simulation of bempedoic acid PK in a virtual adult population were used to test predictions of bioequivalence between the oral suspension and commercial IR tablet. The objective of the current study was to develop a mechanistic PBPK model of bempedoic acid and conduct bioequivalence trials in a virtual population of healthy adult subjects to compare bempedoic acid PK of an oral suspension formulation with the commercial IR tablet.

The physicochemical properties of bempedoic acid confer permeability and solubility within the pH range of the human intestine, resulting in rapid oral absorption and a high fraction of the dose absorbed from the gastrointestinal tract. In vitro passive permeability of bempedoic acid measured in Caco-2 cell monolayers was estimated to be 11.5 × 10^−6^ cm/s. As a weak acid, bempedoic acid was also characterized by pH-dependent aqueous solubility, where aqueous solubility is limited under acidic conditions (pH < 6). Although these physicochemical properties align with BCS class two (low solubility, high permeability) drugs [3], bempedoic acid is much more soluble at pH conditions of the small intestine and is readily absorbed as evidenced by a median time to peak plasma concentration observed at 3.5 h after oral administration [4]. In addition, human mass balance results indicated that a high fraction of the bempedoic acid oral dose was absorbed after a single [^14^C] bempedoic acid 240 mg dose administration, where approximately 95% of the dose was absorbed and <5% of the dose was excreted as unchanged drug [5]. Collectively, these in vitro data and clinical observations suggest that neither bempedoic acid solubility nor permeability are rate-limiting factors in terms of the rate and extent of oral absorption. 

Weak acids, such as nonsteroidal anti-inflammatory drugs [12,13] and warfarin [14] which are all BCS class 2, are insoluble at gastric pH but quickly dissolve at the pH of the intestinal lumen and exhibit linear PK and high oral bioavailability. Moreover, BCS class two drugs, in particular weak acids, turn out to be good candidates for biowaiver consideration when they exhibit good oral absorption despite having low solubility at acidic pH conditions [15,16]. For these weak acid BCS class two drugs and bempedoic acid, the rate-limiting step for absorption is expected to be gastric emptying rather than dissolution [17]. Consistent with these expectations, sensitivity analyses of the final PBPK model showed prediction of bempedoic acid C_max_ was impacted by gastric transit time. We suggested that the application of PBPK modeling could support the pharmaceutical development of an oral suspension formulation of bempedoic acid, given its solubility at pH conditions of the human gastrointestinal tract, consistent with a recent draft FDA guidance [6]. 

## 5. Conclusions

An initial PBPK model was developed which accurately predicted bempedoic acid plasma concentration–time profiles after administration of bempedoic acid as an oral solution or IR tablet. Dissolution data in relevant media were collected for the pilot scale suspension batch and commercial IR tablet formulation under identical conditions and incorporated into the model. Model predictions of oral suspension and IR tablet dissolution were aligned with in vitro dissolution experimental results and sensitivity analyses confirmed a lack of impact resulting from uncertainties associated with physiological estimates of gastrointestinal parameters used in the model. Upon verification of the PBPK final model, virtual trial simulations were conducted to assess the bioequivalence of the oral suspension and IR tablet. Results showed the predicted point estimates and inter-subject variability of the ratio of geometric least square means of the suspension (test) to commercial IR tablet (reference) were within the bioequivalence limits of 80% to 125% for bempedoic acid C_max_ and AUC exposures. Based on the permeability and solubility characteristics of bempedoic acid, dissolution properties of the IR tablet, and corresponding in vivo performance when dosed as an oral solution or IR tablet, the rate and extent of bempedoic acid absorption are unlikely to exhibit meaningful differences when dosed as an oral suspension. Consequently, a bioequivalence study in adults to support the use of the liquid formulation in a pediatric study was not required.

## Figures and Tables

**Table 1:** Summary of Clinical Studies Used in Model Development and Verification.

Clinical Study ^a^	Description	Bempedoic Acid Dose Regimen and PK Data Used to Support Model Development and Verification	Sample Timepoints of Bempedoic Acid Plasma Concentration Determinations
001	Bempedoic acid absorption, distribution, metabolism, and excretion in healthy subjects (n = 6)	Single 240 mg dose as an oral solution formulation. Single-dose plasma PK results were used	Day 1 (pre-dose and 2, 4, 6, 12, 24, 48, 96, 144, and 168 h)
002	Bioequivalence of 2 tablet formulations in healthy subjects (n = 58)	Single 180 mg dose as a tablet. Plasma PK results for the reference formulation (development tablet) of a 2-period crossover were used	Days 1 and 15; reference tablet PK only (pre-dose and 0.5, 1, 2, 3, 4, 6, 8, 10, 12, 24, 48, 72, 96, and 120 h)
003	Drug–drug interaction of steady-state bempedoic acid with concomitant single-dose statin therapy in healthy subjects (n = 48)	Multiple dosing at 180 mg/day. Day 12 steady-state plasma PK results were used	Days 10 and 11 of bempedoic acid dosing (pre-dose) and Day 12 (pre-dose and 0.5, 1, 1.5, 2, 3, 4, 5, 6, 8, 10, 12, 18, 24, 36, 48, 72, 96, and 120 h)
004	Crossover bioequivalence study with 2 tablet formulations in healthy subjects (n = 59)	Single 180 mg dose crossover of commercial (Formulation 2; test) and development (Formulation 1; reference) tablets. Plasma PK results from both groups were used	Days 1 and 15 (pre-dose and 0.5, 1, 2, 3, 4, 6, 8, 10, 12, 24, 48, 72, 96, and 120 h)

PK, pharmacokinetics. ^a^ Study 001 was conducted at Quotient Bioresearch (Northamptonshire, UK), Study 002 was conducted at Covance Clinical Research Unit (Madison, WI, USA), Study 003 was conducted at Jasper Clinic (Kalamazoo, MI, USA), and Study 004 was conducted at BioPharma Services (Toronto, ON, Canada).

**Table 2:** PBPK base model verification—model predictions of bempedoic acid PK parameters compared with observed clinical PK following bempedoic acid 180 mg IR tablet administration. Data presented as mean (±SD) unless otherwise stated.

Bempedoic Acid 180 mg Regimen	Clinical Study	Concomitant Treatment (No. Subjects)	Estimate	C_max_, μg/mL	AUC_inf_ (Single Dose) or AUC_24h_ (Repeat Dose) ^a^, μg·h/mL	t_max_^b^, h
Single Dose	002	NA (N = 58)	Predicted	18.0 (2.60)	273 (102)	1.55 (1.15–3.50)
			Observed	13.5 (3.19)	225 (70.3)	3.00 (1.00–6.00)
			Pred/Obs Ratio	1.33	1.21	0.52
Repeat Dose (Steady-State)	003	Atorva80 (N = 12)	Predicted	24.5 (5.82)	267 (111)	1.60 (1.15–3.50)
			Observed	27.3 (6.98)	348 (95.7)	1.50 (1.00–4.03)
			Pred/Obs Ratio	0.90	0.77	1.07
		Simva40 (N = 12)	Predicted	23.8 (5.46)	260 (105)	1.60 (1.15–3.50)
			Observed	24.7 (6.99)	276 (64.5)	2.00 (1.00–3.00)
			Pred/Obs Ratio	0.96	0.94	0.80
		Prava80 (N = 12)	Predicted	25.1 (6.06)	274 (116)	1.55 (1.15–3.45)
			Observed	23.7 (5.59)	289 (106)	2.00 (1.00–3.00)
			Pred/Obs Ratio	1.06	0.95	0.78
		Rosuva40 (N = 12)	Predicted	24.3 (5.76)	265 (108)	1.60 (1.15–3.50)
			Observed	21.9 (9.60)	264 (129)	1.75 (1.00–4.00)
			Pred/Obs Ratio	1.11	1.01	0.92

Atorva80, atorvastatin 80 mg; AUC_24h,_ area under the concentration–time curve from time zero to 24 h; AUC_inf_, area under the concentration–time curve from time zero to infinity; C_max,_ maximum concentration; IR, immediate release; NA, not applicable; PBPK, physiologically based pharmacokinetic; PK, pharmacokinetics; Prava80, pravastatin 80 mg; Rosuva40, rosuvastatin 40 mg; SD, standard deviation; Simva40, simvastatin 40 mg; t_max,_ time to maximum concentration. ^a^ AUC_inf_ after single-dose administration in Study 002 or AUC_24h_ at steady state in Study 003. ^b^ Median (range).

**Table 3:** Input parameters of final PBPK model.

Parameter	Estimate	Comment or Parameter Source
Molecular weight	344.5	Internal data on file
Ionization	Diprotic acid	Internal data on file
pKa_1_	4.88	Internal data on file
pKa_2_	5.60	Internal data on file
Intrinsic solubility, mg/mL	0.0051	Internal data on file
Log P	4.328	Internal data on file
DLM scalar (tablet/suspension)	0.07/0.3	Model-defined parameter
Caco-2 permeability, ×10^−6^ cm/s	11.5	Internal data on file
Permeability estimate, ×10^−4^ cm/s	3	Model-defined parameter
Tablet, particle radius, µm	18.2	Internal data on file (D50 measured: 36.4 µm) ^a^
Suspension, particle radius, µm	25	Internal data on file (D50 measured: 50 µm)
Suspension, bempedoic acid, mg/mL	20	Internal data on file
Suspension, drug fraction dissolved	0.01%	Model-defined parameter
Suspension, viscosity, cps	118.8	Internal data on file
Blood:Plasma ratio	0.55	Study 1002-001
Plasma unbound fraction	0.026	Internal data on file
f_a_	0.97	Study 1002-001
f_g_	1.0	100% gut availability assumed
K_p_ scalar	2	Model-defined parameter
V_sac_, L/kg	0.1	Model-defined parameter
CL_in_ (L/h)/CL_out_, L/h	3.16/1.32	Model-defined parameters
V_ss_, L/kg	0.14	Model-defined parameter
CL_oral_, L/h	0.81	Study 1002-001
CL_renal_, L/h	0.03	Study 1002-001
CL_u,int,H_, µL/min/mg protein	8.17	Model-defined parameter

CL_in_, input clearance; CL_out_, output clearance; CL_oral_, oral clearance; CL_renal_, renal clearance; CL_u,int,H_, unbound hepatic intrinsic clearance; cps, centipoise; DLM, diffusion layer model; D50, median diameter; f_a_, fraction of dose absorbed; f_g_, gut availability; K_p_, plasma partition coefficient; PBPK, physiologically based pharmacokinetic; V_sac_, single adjusting compartment volume; V_ss_, steady-state distribution volume. ^a^ Midpoint of 31.6 µm to 41.2 µm D50 range.

**Table 4:** Correlation coefficients for bempedoic acid C_max_, t_max_, and AUC_inf_ predictions relative to changes in gastrointestinal physiology parameters from simulations of 10 virtual trials.

Gastrointestinal Physiology Parameter	C_max_	t_max_	AUC_inf_
Gastric TT	−0.32	0.40	0.07
Stomach pH (fasted)	0.00	−0.02	−0.01
Small Intestine TT	−0.09	0.17	−0.02
Colon TT	−0.11	0.00	−0.12

AUC_inf_, area under the concentration–time curve to infinity; C_max_, maximum concentration; t_max_, time to maximum concentration; TT, transit time (mean residence time, h) in a given gastrointestinal segment.

**Table 5:** Final model bempedoic acid PK parameter predictions and ratios of geometric least square mean estimates (90% CI) of bempedoic acid PK parameters predicted for 180 mg doses of bempedoic acid formulated as a pilot scale suspension (Test) and commercial tablet (Reference) by crossover and parallel study designs.

Virtual Study Design	Parameter	Pilot Scale Suspension GM Estimate (%CV)	Commercial Tablet GM Estimate (%CV)	Test/Reference Ratio (90% CI)
Crossover	C_max_, µg/mL	19.6 (11.5)	19.6 (12.7)	99.7% (96.1–103)
	t_max_, h	1.57 (38.0)	1.56 (38.3)	nc
	AUC_last_, µg·h/mL	313 (33.2)	313 (33.2)	100% (90.6–110)
	AUC_inf_, µg·h/mL	329 (39.2)	329 (39.2)	100% (89.1–112)
Parallel	C_max_, µg/mL	17.2 (13.0)	17.8 (16.8)	96.9% (92.6–101)
	t_max_, h	1.85 (34.2)	1.68 (36.3)	nc
	AUC_last_, µg·h/mL	259 (38.8)	264 (41.4)	98.2% (87.3–111)
	AUC_inf_, µg·h/mL	270 (42.6)	274 (45.9)	98.4% (86.4–112)

AUC_inf,_ area under concentration–time curve from time zero to infinity; AUC_last,_ area under concentration–time curve from time zero to last timepoint; CI, confidence interval; C_max_, maximum concentration; CV, coefficient of variation; GM, geometric mean; nc, not calculated; PK, pharmacokinetics; t_max_, time to maximum concentration.

## Data Availability

The data, analytical methods, and study materials will not be made available to other researchers for the purposes of reproducing the results or replicating the procedures due to privacy or ethical restrictions.

## Supplementary Materials

The following supporting information can be downloaded at: https://www.mdpi.com/article/10.3390/pharmaceutics15051476/s1, Figure S1: Bempedoic acid physiologically based pharmacokinetic (PBPK) model schematic; Table S1: Summary of bempedoic acid pharmacokinetic parameters after single 180 mg bempedoic acid administration and statistical analysis for the comparison of formulation 2 to formulation 1 (Study 004); Table S2: Dissolution of bempedoic acid 180 mg immediate release tablets; Table S3: Dissolution of bempedoic acid 180 mg oral suspension (20 mg/mL).

## Abbreviations

ACSVL1, very long-chain acyl-CoA synthetase 1; ADAM, advanced dissolution absorption and metabolism; AUC, area under the concentration–time curve; AUC_24h_, AUC from time zero to 24 h; AUC_inf_, AUC from time zero to infinity; AUC_last_, AUC from time zero to last quantified concentration; BCS, biopharmaceutical classification system; CI, confidence interval; CL, clearance; C_max_, maximum concentration; DDI, drug–drug interaction; DLM, diffusion layer model; FDA, US Food and Drug Administration; HCl, hydrochloric acid; HeFH, heterozygous familial hypercholesterolemia; HPLC, high-performance liquid chromatography; IR, immediate release; LC-MS/MS, liquid chromatography-tandem mass spectrometry; LDL-C, low-density lipoprotein cholesterol; PBPK, physiologically based pharmacokinetics; PK, pharmacokinetics; t_max_, time to maximum concentration; UGT, uridine 5’ diphospho-glucuronosyltransferase; V_ss_, volume of distribution at steady state.
