# Supplementary material for: Physiologically Based Pharmacokinetic Model Development and Verification for Bioequivalence Testing of Bempedoic Acid Oral Suspension and Reference Tablet Formulation
Source: Pharmaceutics. 2023 May 12;15(5):1476. doi: 10.3390/pharmaceutics15051476 (PMC10222242; doi:10.3390/pharmaceutics15051476)
Supplement: Supplementary file 1 [file pharmaceutics-15-01476-s001.zip › pharmaceutics-2325274-supplementary.pdf]

pharmaceutics

MDPI

Supplementary Materials: Physiologically Based Pharmacokinetic Model Development and Verification for Bioequivalence Testing of Bempedoic Acid Oral Suspension and Reference Tablet Formulation

Figure S1. Bempedoic acid physiologically based pharmacokinetic (PBPK) model schematic.

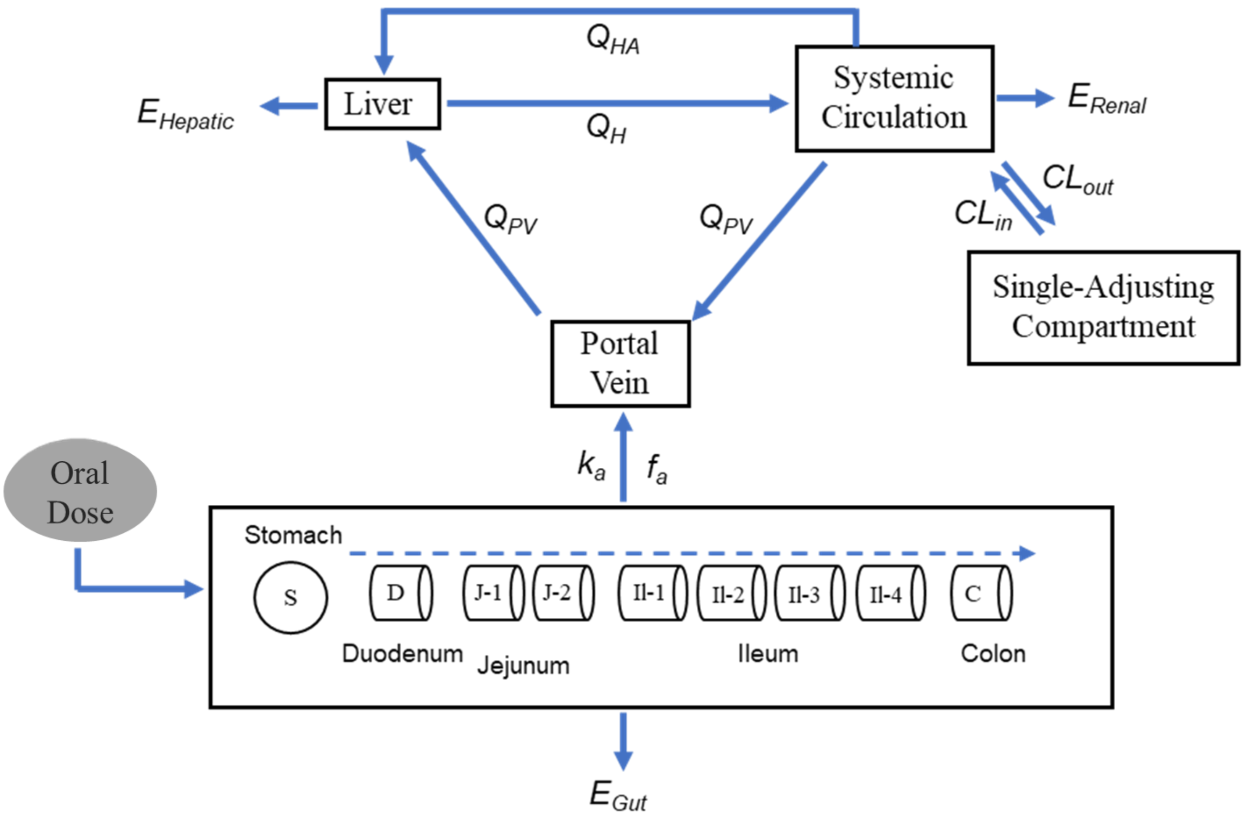

CL_{in}, CL_{out}, intercompartmental clearance; C, colon; D, duodenum; E_{Gut}, $E_{Hepatic}$, E_{Renal}, extraction by the gut, liver and kidney, respectively; f_a, fraction of dose absorbed; Il, ileum; J, jejunum; k_a, absorption rate constant; Q_H, Q_{HA}, Q_{PV}, blood flow in liver, hepatic artery and portal vein, respectively; S, stomach.

Table S1. Summary of bempedoic acid pharmacokinetic parameters after single 180 mg bempedoic acid administration and statistical analysis for the comparison of formulation 2 to formulation 1 (Study 004).

PK Parameter	Geometric LS Mean		Ratio of Geometric LS Means (90% CI)
	Formulation 2 (n=59)	Formulation 1 (n=58)	Formulation 2 to Formulation 1
C_{max}, μg/mL	18.4	18.7	0.983 (0.9421–1.0263)
AUC_{inf}, μg·h/mL	269	265	1.016 (0.9876–1.0454)
AUC_{last}, μg·h/mL	263	260	1.012 (0.9855–1.0398)

AUC_{inf}, area under the concentration-time curve from time zero to infinity; AUC_{last}, area under concentration-time curve from time zero to last timepoint; CI, confidence interval; C_{max}, maximum concentration; LS, least square; PK, pharmacokinetics. C_{max}, AUC_{last}, AUC_{inf} estimates are rounded to 3 significant figures.

The log-transformed PK parameters (AUC_{last}, AUC_{inf} and C_{max}) were analyzed using a linear mixed model, with fixed effects for treatment, period and sequence, subject within sequence as random effect.

Formulation 1, immediate release tablet used during clinical development; Formulation 2, commercial immediate release tablet.

Table S2. Dissolution of bempedoic acid 180 mg immediate release tablets.

Buffer	Average % Dissolved (Released), n=12							
	5 min	10 min	15 min	20 min	30 min	45 min	60 min	75 min
0.1N HCl pH 1.2	0	1	1	2	2	2	3	3
50 mM Acetate pH 4.5	0	3	3	4	5	5	5	5
50 mM Phosphate pH 6.6	26	69	93	99	101	101	101	101
50 mM Phosphate pH 6.8	33	82	101	103	104	104	104	104

Dissolution of bempedoic acid tablet (Lot 99743-07D) was determined using USP apparatus II operated at 50 rpm in 900 mL media volume.

Table S3. Dissolution of bempedoic acid 180 mg oral suspension (20 mg/mL).

Buffer	Average % Dissolved (Released), n=12							
	5 min	10 min	15 min	20 min	30 min	45 min	60 min	75 min
0.1N HCl pH 1.2	2	2	2	2	3	3	3	3
50 mM Acetate pH 4.5	2	3	4	4	4	4	4	4
50 mM Phosphate pH 6.6	75	87	90	91	92	93	93	96
50 mM Phosphate pH 6.8	88	91	92	92	93	93	94	95

Dissolution of bempedoic acid suspension formulation (Lot 0000091928) was determined using USP apparatus II operated at 50 rpm in 900 mL media volume.
